# Pseudoreplication and greenhouse-gas emissions from rivers

**DOI:** 10.1038/s41467-019-13303-1

**Published:** 2019-11-26

**Authors:** Scott D. Tiegs, Thomas Raffel

**Affiliations:** Department of Biological Sciences, Oakland University, Rochester, MI USA 48309

**Keywords:** Carbon cycle, Carbon cycle, Freshwater ecology

**Arising from** S. Comer-Warner et al. *Nature Communications* 10.1038/s41467-018-04756-x

Rivers are hotspots of microbially derived greenhouse-gas emissions (*sensu* McClain^1^) and so it is critical to determine how microbial activity depends on environmental factors such as temperature and geology. The recently published experiment of Comer-Warner^2^ addresses these and related topics, and the authors conclude that microbial responses to warming are non-linear, have a threshold response, and vary with geology, sediment size, and organic-matter content. Unfortunately, these conclusions were founded on badly pseudoreplicated experimental designs (*sensu* Hurlbert^3^), undermining the generality of the results. We therefore urge readers to be cautious about accepting the general conclusions presented in the article, and suggest that follow-up studies should be a priority for the broader research community to determine if these results are repeatable in other lab or field environments.

The research presented by Comer-Warner^2^ contains multiple methodological flaws that undermine their conclusions and the degree to which they can be generalized. Some of these are procedural weaknesses that were given a small amount of attention in the article (e.g., potential artefacts caused by holding microbial field samples for weeks at low temperatures). Others, however, are more-serious experimental-design flaws that were not addressed and limit the generalizations that one can reasonably deduce from the results. Here we focus on this latter problem due to the broad generality of conclusions drawn by the authors, which went beyond the sphere of statistical inference their experimental approach allowed.

The first design flaw we highlight is the attribution of geological effects even though there was zero replication of geology as a treatment. Their experiment involved gathering sediment samples from two rivers: one river flowing over calcareous geology and the other flowing over sandstone. Sediments were then stored in the lab at low temperature (4 °C) for several weeks, potentially introducing important context dependencies due to changes in the microbial community during this time (as acknowledged by the authors). This design allows them to conclude with some degree of confidence that there are differences in microbial activity between the two rivers. Importantly, however, the reasons why there are differences between (e.g., geology) them cannot be stated with any degree of confidence given this lack of replication. A more robust experiment to test this hypothesis would involve gathering sediment from several separate rivers flowing over each bedrock type, and selecting sampling locations to maximize treatment interspersion and help control for spatial confoundment.

The second design flaw we note is that temperature treatments were incorrectly replicated. The authors appear to have made use of a single incubator, repeating the same experiment each week at a different temperature. They randomized the order of temperature treatments, a design decision that reduced temporal confoundment, but importantly, does not address potentially greater problems associated with isolative segregation (after Hurlbert^3^). For any given temperature treatment in a given week, all of the cultures were incubated together as a batch; i.e., the temperature treatment itself was pseudoreplicated. Cultures from a given temperature treatment experienced common batch-specific conditions that likely differed in unknown ways from batches tested in other weeks, and therefore cannot be treated as independent from each other—a key assumption of the inferential statistics employed by the authors. It is plausible that random effects of week could explain much of the observed among-temperature variation, including the suspiciously erratic shifts that sometimes occurred from one temperature to the next. As a result, we are skeptical of the conclusions regarding both non-linear and threshold temperature effects—key messages of the paper—both of which are founded largely on the observed reduction in microbial activity over one of the four temperature intervals examined, from 21 to 26 °C. Furthermore, these results warrant some degree of skepticism because they contradict linear temperature effects observed by prior studies across similar ranges of temperatures (e.g., see the meta-analysis of Yvon-Durocher et al.^4^).

In aggregate, the experimental design flaws in Comer-Warner et al.^2^ strongly limit the scope of inferences that can reasonably be drawn from their results. These sorts of design flaws were explored in detail in the paper of Hurlbert^3^, which has since served as a framework and a reference for robust design and analysis of natural and manipulative experiments in the ecological and environmental sciences. The research questions addressed by Comer-Warner et al. are important and needed. However, more-rigorous experiments are required before broad conclusions can reasonably be drawn about the influence of warming and geology on greenhouse-gas emissions from stream ecosystems.

## References

[1] McClain M (2003). Ecosystems.

[2] Comer-Warner S (2018). Thermal sensitivity of CO_2_ and CH_4_ emissions varies with streambed sediment properties. Nat. Commun..

[3] Hurlbert SH (1984). Pseudoreplication and the design of ecological field experiments. Ecol. Monogr..

[4] Yvon-Durocher G (2014). Methane fluxes show consistent temperature dependence across microbial to ecosystem scales. Nature.

